# Polymorphisms in leucine-rich repeat genes are associated with autism spectrum disorder susceptibility in populations of European ancestry

**DOI:** 10.1186/2040-2392-1-7

**Published:** 2010-03-25

**Authors:** Inês Sousa, Taane G Clark, Richard Holt, Alistair T Pagnamenta, Erik J Mulder, Ruud B Minderaa, Anthony J Bailey, Agatino Battaglia, Sabine M Klauck, Fritz Poustka, Anthony P Monaco, International Molecular Genetic Study of Autism Consortium (IMGSAC)

**Affiliations:** 1 Wellcome Trust Centre for Human Genetics, University of Oxford, Oxford OX3 7BN, UK; 2 Departments of Epidemiology and Public Health, and Infectious and Tropical Diseases, London School of Hygiene and Tropical Medicine, London WC1E 7HT, UK; 3 Department of Psychiatry, Child and Adolescent Psychiatry, University Medical Center Groningen, PO Box 660, 9700 AR Groningen, the Netherlands; 4 University Department of Psychiatry, Warneford Hospital, Oxford, UK; 5 Stella Maris Clinical Research Institute for Child and Adolescent Neuropsychiatry, Calambrone (Pisa), Italy; 6 Division of Molecular Genome Analysis, German Cancer Research Center, Heidelberg, Germany; 7 Department of Child and Adolescent Psychiatry, Johann Wolfgang Goethe-University, Frankfurt/Main, Germany

## Abstract

**Background:**

Autism spectrum disorders (ASDs) are a group of highly heritable neurodevelopmental disorders which are characteristically comprised of impairments in social interaction, communication and restricted interests/behaviours. Several cell adhesion transmembrane leucine-rich repeat (LRR) proteins are highly expressed in the nervous system and are thought to be key regulators of its development. Here we present an association study analysing the roles of four promising candidate genes - *LRRTM1 *(2p), *LRRTM3 *(10q), *LRRN1 *(3p) and *LRRN3 *(7q) - in order to identify common genetic risk factors underlying ASDs.

**Methods:**

In order to gain a better understanding of how the genetic variation within these four gene regions may influence susceptibility to ASDs, a family-based association study was undertaken in 661 families of European ancestry selected from four different ASD cohorts. In addition, a case-control study was undertaken across the four LRR genes, using logistic regression in probands with ASD of each population against 295 ECACC controls.

**Results:**

Significant results were found for *LRRN3 *and *LRRTM3 *(*P *< 0.005), using both single locus and haplotype approaches. These results were further supported by a case-control analysis, which also highlighted additional SNPs in *LRRTM3*.

**Conclusions:**

Overall, our findings implicate the neuronal leucine-rich genes *LRRN3 *and *LRRTM3 *in ASD susceptibility.

## Background

Autism is a genetically complex neurodevelopmental disorder, characterized by impairments in reciprocal social interaction and communication, along with restricted and stereotyped patterns of interests and behaviours [1]. It is an extremely heterogeneous and highly heritable condition, affecting predominantly males (with an average sex ratio of 4:1), and with an onset before 3 years of age [2,3]. Autism spectrum disorders (ASDs) refers to a broad definition of autism, including classical and atypical autism, Asperger syndrome and pervasive developmental disorder not otherwise specified [4]. The aetiology of ASD is not fully understood and the causal variants and their modes of transmission remain elusive.

Leucine-rich repeats (LRRs) are common protein-protein interaction motifs and are typically 20-29 amino acids in length [5]. LRR proteins are highly expressed in the nervous system and are involved in numerous biological functions, including nervous system development [5-10]. Mutations in LRR genes have been associated with different diseases, such as hereditary lateral temporal epilepsy [11] and Parkinson disease [12]. Furthermore, a recent study suggested that LRR variants could possibly be involved in ASD susceptibility [13].

From the 313 known human LRR genes (NCBI Build 36.1), this work focused on four brain-enriched LRR candidates - *LRRTM1*, *LRRTM3*, *LRRN1 *and *LRRN3*. There are several LRR subfamilies, differentiated by the consensus sequence of the repeat and/or different combinations of supplementary domains [5,8,14]. One family of brain-enriched LRR containing type I transmembrane proteins is termed the LRRTM (leucine rich repeat transmembrane neuronal) family [8]. This is a highly conserved four-member family which, with the exception of *LRRTM4*, has the unusual characteristic of being located in the introns of different α-catenin genes. Catenin family members are adhesion proteins that can form a complex with cadherins, which themselves have also been implicated in intellectual disability, autism and ASD risk [13,15-17]. LRRTM messenger (m)RNAs are mainly expressed in the nervous system, each with a distinct and highly regulated pattern [8,18]. It has recently been proposed that LRRTMs are synaptic organizing molecules and synaptogenic inducers in neurons, initiating excitatory presynaptic differentiation and mediating post-synaptic specializations [19]. As several synaptic genes have been implicated in ASD, these genes become promising candidates to study [20].

*LRRTM1 *(MIM*610867), located on 2p12, lies within intron 7 of *CTNNA2 *(*α2-catenin*) with the highest mRNA expression in the brain and salivary gland [8]. *LRRTM1 *is the first gene to be associated with both human handedness (relative hand skill) and schizophrenia [21] and is thought to be involved in brain development, neuronal connectivity, intracellular trafficking in axons and synaptogenesis [19,21]. Therefore, it is an interesting candidate for autism where there is already some evidence for associations with abnormal asymmetrical brain structure in language-associated areas [22,23].

Similarly, *LRRTM3 *(MIM*610869), on 10q22.1, is positioned within intron 7 of *CTNNA3 *(*αT-catenin*). This gene has a more restricted expression profile compared to *LRRTM1*, with expression in the brain, particularly the cerebellum [8,24]. In addition, *LRRTM3 *is functionally and positionally linked to late-onset Alzheimer's disease [24,25]. Moreover, *CTNNA3 *has also been recently suggested to be associated to ASD susceptibility [13].

The LRRN (leucine rich repeat neuronal) family of proteins has four known members in humans, all being brain-enriched type I transmembrane proteins [26]. Studies in several species show that the members of this family have different spatial and temporal expression patterns and that they are involved in neural development and regeneration [14,27-29]. Although they seem to function as adhesion molecules or binding receptors in regulatory mechanisms, their biological activities and specific central nervous system (CNS) functions in humans are still unclear [30,31].

*LRRN1*, located on 3p26.2, is again a nested gene located in intron 8 of the long form of *SUMF1 *(*sulphatase modifying factor 1*) (NM_182760). In the developing neural cells of the chick, Lrrn1 is localized in the endosome, suggesting that it might be involved in the regulation of cell adhesion and signalling pathways [32]. *LRRN1 *shows a high degree of sequence conservation and comparison to its expression in the mouse indicates that it is dynamically expressed in the somites and the neural plate during development, and mostly in the brain and kidney in the adult [14]. Furthermore, *LRRN1 *is located within a candidate region for recessive non-syndromic mental retardation on chromosome 3 [33]. Additionally, a paternally inherited duplication (3p26.1-26.2) which encompasses *LRRN1 *was reported in children with autism and additional developmental abnormalities [34].

Finally, *LRRN3 *maps to 7q31.1 and is brain-enriched [14,31,35]. *LRRN3 *is a nested gene in *IMMP2L *(*inner mitochondrial membrane peptidase-like*), being positioned within intron 3. The molecular structure and expression pattern of *Lrrn3*, suggest that it plays a role in the development and maintenance of the nervous system [14]. Other studies have shown that *Lrrn3 *exhibits regulated expression in the developing ganglia and motor neurons of the neural system, and is upregulated during neuronal cortical injury [14,27,28]. In addition, one of the most consistently replicated loci for autism, first identified by The International Molecular Genetic Study of Autism Consortium (IMGSAC) [36], is on 7q21.3-7q34 [37-43]. As *LRRN3 *is located under the linkage peak of interest for IMGSAC families, and there is evidence of an association with the *IMMP2L/DOCK4 *region in the IMGSAC cohort [44], we considered *LRRN3 *to be an appealing candidate for ASD susceptibility, despite a previous study failing to find evidence of an association between this gene and autism in their cohorts [45].

Here, the roles of *LRRTM1*, *LRRTM3*, *LRRN1 *and *LRRN3 *in ASD susceptibility were studied in four different populations of European ancestry (IMGSAC, Italian, German and Northern Dutch). The variation at these four loci was assessed and the association of gene variants and haplotypes with ASD was tested in a family based study. Significant evidence of their association to ASD was found for *LRRN3 *and *LRRTM3*. In order to complement this analysis, a case-control study was performed, partially supporting the evidence of association found in *LRRN3 *and *LRRTM3*. To our knowledge, this is one of the most comprehensive genetic analyses of association between these genes and ASD risk.

## Methods

### Subjects

All the individuals who participated in this study are Caucasian and of European ancestry. Four cohorts were used, our core IMGSAC set consisting mainly of UK families, two groups from Italy and Germany (collected as part of the IMGSAC collaboration) and a population from the north of the Netherlands. These cohorts are referred to as IMGSAC, Italian, German and Northern Dutch, respectively. A total of 2758 individuals from 661 families [439 IMGSAC (350 multiplex and 89 singleton), 85 Italian, 30 German and 107 Northern Dutch] were analysed. The male:female ratio of the affected individuals is 4.1:1. Also, a cohort of 295 UK DNA controls [European Collection of Cell Cultures (ECACC]) was used for the case-control study. ECACC controls were randomly selected, non-related, UK Caucasian blood donors http://www.hpacultures.org.uk/products/dna/hrcdna/hrcdna.jsp. For the IMGSAC sample collection the identification of families, assessment methods and inclusion criteria used have been described previously [37]. Briefly, after identification in an initial screen for autism, parents undertook the Autism Diagnostic Interview-Revised (ADI-R) [46] and the Vineland Adaptive Behaviour Scales [47]. Probands were assessed using the Autism Diagnostic Observation Schedule (ADOS) [48] and a clinical evaluation was undertaken in order to exclude known medical disorders aetiologically associated with autism (for example, tuberous sclerosis and neurofibromatosis). Karyotypes were obtained for probands when possible and molecular genetic testing for fragile X syndrome carried out on one affected case per family. A blood sample was taken from both probands and available first degree relatives. DNA was extracted from blood samples, buccal swabs or cell lines using a DNA purification kit (Nucleon^® ^BACC2 Blood and Cell Culture DNA purification kit, Tepnel Life Sciences, Manchester, UK) and standard techniques. The Italian and German populations had the same assessment as that described above as they are part of the wider IMGSAC group. For the Northern Dutch population the subjects and their assessment are again described in detail elsewhere [49]. In short, the families were recruited through an epidemiological survey and an autism outpatient clinic affiliated with the Child and Adolescent Psychiatry Center of Groningen. The diagnosis criteria were very similar to those previously described for IMGSAC (including ADI-R and ADOS assessments) and the children's intellectual functioning was evaluated as described by Mulder *et al*. [50]. DNA was extracted from cheek cells obtained by mouth swabs from patients, parents and siblings using the Epicentre MasterAmp™ DNA Extraction Solution Kit (BiozymTC, Landgraaf, the Netherlands). The study was reviewed by the relevant ethical committees. For IMGSAC population cohort the ethical approval was carried out by:

(a) Europe (*UK *- Oxfordshire Psychiatric Research Ethics Committee A, under the review number O03.013 and title name 'Identifying and understanding the actions of autism susceptibility genes'; Cambridge Local Research Ethics Committee; Institute of Psychiatry Ethical Research Committee; Joint Ethics Committee (Newcastle & North Tyneside Health Authority/Universities of Newcastle upon Tyne/Northumbria); Salford and Trafford Research Ethics Committee/*Denmark *- Den Videnskabsetiske Komite, Kobenhavns Amt/*Finland *- Ethical Committee for the Hospital of Children and Adolescents and Psychiatry (University Central Hospital)/*France *- Comité Consultatif de Protection des Personnes dans la Recherche Biomédicale Groupe Hospitalier Pitié-Salpêtrière-Paris/*Germany *- Ethik-Kommission des Fachbereichs Medizin der Johann Wolfgang Goethe (Universität Frankfurt am Main); Heidelberg Ethikkommission der Medizinischen Fakultät/*Greece *- Agia Sophia Hospital Ethics Committee/*Italy *- Comitato Etico IRCCS Fondazione Stella Maris/*Netherlands *- Medisch Ethische Toetsingscommissie/*Sweden *- Ethical Review Board of Sahlgren's Academy, Goteborg University Faculty of Medicine

(b) USA - Yale University School of Medicine Human Investigation Committee; University of Illinois at Chicago's Office for the Protection of Research Subjects; University of Michigan Medical School IRBMED

(c) Canada - The Hospital for Sick Children, Research Ethics Board; Hamilton Health Sciences/McMaster University Research Ethics Board.

For the Northern Dutch population cohort, ethical review was done by the Medical Ethical Committee of the University Medical Center Groningen under review number M08.057360.

Written informed consent was provided by all parents/guardians and, when possible, by the affected individuals. The IMGSAC cases are Caucasian, but from different sites in Europe and the USA. Therefore, population substructure is a distinct possibility. The samples used for the current study have substantial overlap with the IMGSAC samples described in Maestrini *et al*. [44], where no evidence of strong population stratification was detected. However, it is possible that subtle or low levels of population stratification may be present, potentially leading to false positives in the case-control analysis.

### Genotyping

#### Single nucleotide polymorphism (SNP) selection

Genotyping data from Centre d'Etude du Polymorphisme Humain individuals was downloaded from the HapMap phase II (release 24) for each gene and approximately 5 kb of upstream and downstream sequence. This resulted in information for the four following regions being obtained: 2p12 (80381256-80386255); 10q22.1 (68353045-68532626); 3p26.2 (3814162-3866687); and 7q31.1 (110516705-110554408). Sixty-seven haplotype tagging SNPs were chosen across these loci using Tagger from Haploview v4.0 [51] [*r*^2 ^> 0.8 and minor allele frequency (MAF) > 0.05, aggressive tagging], capturing the maximum amount of genetic variation in the four regions. In addition, one 3'untranslated region and two synonymous SNPs in *LRRTM1 *were genotyped, as these were the only markers left to genotype in order to complete the whole coverage of this region.

#### Sequenom assay

The genotyping assay was designed using the MassARRAY software (Sequenom) and genotypes obtained using the MassARRAYTyper™ system (version 3.1.4.0). Quantification of DNA samples was performed using the Picogreen^®^dsDNA Quantitation Kit (Invitrogen, Oregon, USA) and 40 ng of genomic DNA per sample was used in the genotyping assay. Genotyping was performed using the Sequenom MALDI-TOF iPLEX platform (Sequenom, San Diego, USA). The sample and marker genotyping success rates were ~98% and 94%, respectively. Based on two control samples included on each plate as genotyping controls, we found high inter-plate reproducibility (99.8%). Genotype Analyzer (Sequenom) was used to visually check the quality of each genotype call, assigning alleles where possible. An in-house database [52] was used to store all genotypic data and to produce files for statistical analysis.

### Statistical analysis

#### Error checking

Genotypes were checked for Mendelian consistency using PedCheck [53] and any inconsistent genotypes were removed. Genotypes flanking double recombinants were detected using MERLIN [54] and, in ambiguous cases where probable excess recombination occurred, genotypes were reconfirmed and corrected as necessary. All the SNPs were tested for Hardy-Weinberg equilibrium using a χ^2 ^test, in parents and probands separately.

#### Association analysis

**Figure 1:**
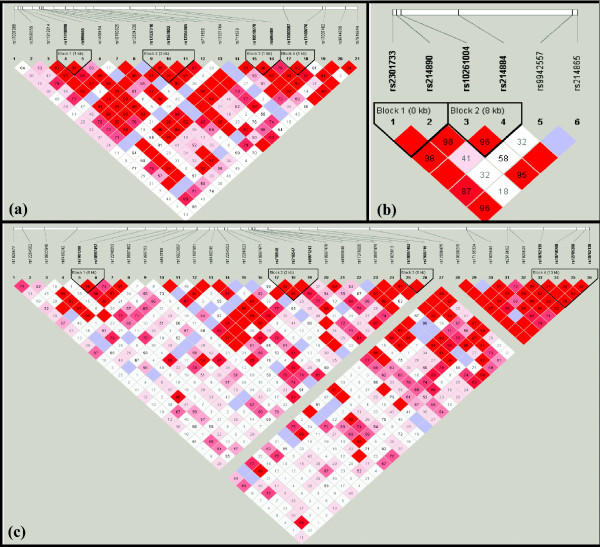
**The graphical output from Haploview**. The markers tested and the haplotype blocks constructed for (a) *LRRN1*, (b) *LRRN3 *and (c) *LRRTM3 *are included. D' values are indicated (bright red corresponds to D' = 1, with the colour tending towards white as D' tends towards 0).

The pairwise linkage disequilibrium (LD) maps for *LRRN1*, *LRRN3 *and *LRRTM3 *were constructed from genotypes using the Haploview software (Figure 1). SNP and haplotype association in families was assessed using the transmission disequilibrium test (TDT) [55], with a version robust to non-independent siblings implemented [56], allowing for the use of multiple siblings within a nuclear family. Allele frequencies were reported for all the parents and children. From applying the TDT, the allele transmission frequencies from parents to offspring were also estimated. Parental transmissions were also analysed for each SNP to consider parent-of-origin effects. In order to combine the evidence across all cohorts at each polymorphism, we estimated odds ratios (ORs) and their standard errors from transmission frequencies [57] and then performed a meta-analysis pooling using an inverse variance approach [58]. Also, at each polymorphism we performed a chi-square test of heterogeneity of the ORs [58]. Meta-analysis forest plots were constructed for each marker using the *meta *library in the R statistical software http://www.r-project.org. A haplotype-based TDT analysis was also carried out using TRANSMIT [56]. For each haplotype, risk estimates and their 95% credibility internals were estimated using a Bayesian method [55,56]. Logistic regression was used in the analysis of both the alleles and genotypes in the case-control study. When we were analysing cases from the same family, we performed a weighted logistic analysis where each family contributed the same weight. In the logistic regression framework, testing on the genotypes (for example, dd, DD and Dd) without regard to any 'order', allelic count or allelic pairing was performed. In particular, we investigated several related genotypic mechanisms: (i) an additive model which assesses the influence of increasing the number of minor alleles (0,1,2) on log-risk; (ii) dominant model (of the 'd' allele: dd/Dd versus DD); (iii) recessive model (of 'd': dd versus Dd/DD); (iv) heterozygous advantage (Dd versus DD/dd); (v) general model (dd versus DD, Dd versus DD). We report the minimum *P*-value from these correlated tests. Meta-analytic approaches were also applied to pool the resulting allele- and genotype-based ORs across populations.

#### Multiple testing correction

Performing multiple statistical tests leads to inflation in the occurrence of false positives and it is therefore necessary to adjust the statistical significance (*P*-value) threshold (usually 5%) in order to account for the number of independent tests. A Bonferroni correction using the total number of SNPs would be too conservative because of the high LD between SNPs in the analysed regions [56]. By considering the LD pattern, which identified 10 independent haplotype blocks, it is reasonable to interpret using a *P *< 0.005 (= 0.05/10) to be statistically significant. Using a separate permutation approach we found a similar (5%) significance threshold (*P *< 0.0048) based on inference of the maximum chi-square statistic observed over all genotyped SNPs. In particular, both the transmitted/untransmitted and case-control status of the chromosomes were randomly permuted 10,000 times. From each of the 10,000 random experiments, in both trio and case-control studies, we determined the maximum chi-square statistics over all SNPs genotyped. We ordered these statistics and then calculated the 95 percentile. This was the estimate of the 0.05 significance level for the experiment performed, assuming inference is taken with respect to maximum chi-square statistic observed over all genotyped SNPs.

## Results

### Family-based studies

**Figure 2:**
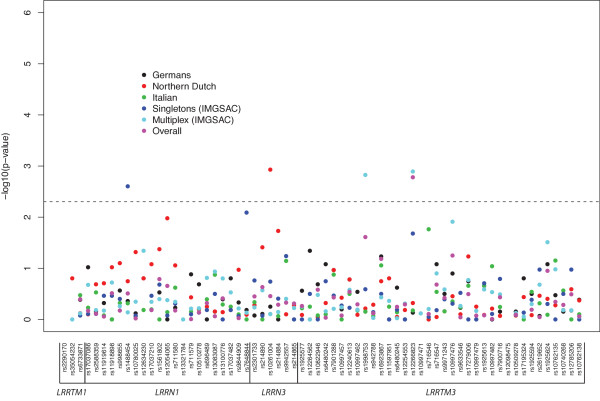
**Transmission disequilibrium test results for each population and overall meta-analysis results, across the four genes under study**. The significance is represented on the *y*-axis, plotted as -log10(*P*-value), and on the *x*-axis is shown each marker studied. The dashed line represents the statistical significance threshold of 0.005.

**Figure 3:**
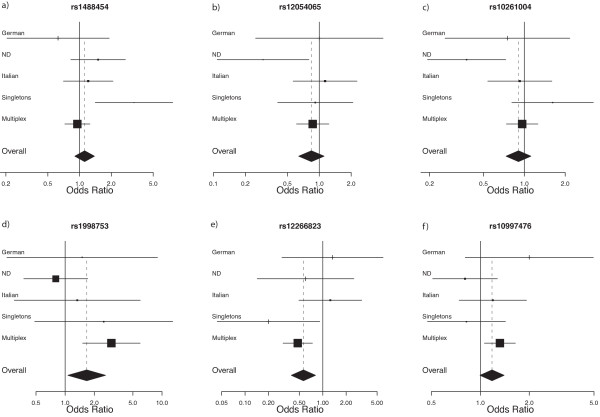
**Forest Plots of the six most significant markers from the transmission disequilibrium test (TDT) analyses**. (a) rs1488454 (*LRRN1*); (b) rs12054065 (*LRRN1*); (c) rs10261004 (*LRRN3*); (d) rs1998753 (*LRRTM3*); (e) rs12266823 (*LRRTM3*); and (f) rs10997476 (*LRRTM3*). For each marker, the results for each population and for the overall meta-analysis TDT are represented. The population's labels correspond respectively to: German, ND (Northern Dutch), Italian, singleton families (IMGSAC) and multiplex families (IMGSAC). For each marker, the odds ratio (OR) is represented on the *x*-axis, and on the *y*-axis is shown the point estimate and its 95% confidence interval (CI). The overall meta-analysis estimate and its CI are at the bottom, represented as a diamond (indicating the pooled point estimate). The size of the black square represents the amount of data analysed for each population. The vertical bold line shows the no effect point (OR = 1) and the dotted line shows the overall effect point.

Sixty-six markers (from the 70 in total) were successfully genotyped across these *loci*. One marker in *LRRTM1 *(rs34285492) and three in *LRRTM3 *(rs1925574, rs1925575, rs6480244) failed and were removed from the analysis. For the remaining markers, genotype and allele frequencies were calculated from cases and parents for each population across the genes under study. Furthermore, two markers - rs2290170 (*LRRTM1*) and rs12098475 (*LRRTM3*) - were monomorphic in the populations studied. The SNPs analysed were in Hardy-Weinberg equilibrium in both probands and founders (*P *> 0.001). TDT statistics were performed for all markers in each population to test for association between the four leucine-rich repeat (LRR) genes and ASD, and it was considered reasonable to interpret a *P *< 0.005 to be statistically significant (see Materials and Methods). For the IMGSAC families, the analysis was carried out as two groups, since both multiplex and singleton families were present. This follows the suggestions that these two groups have different underlying aetiologies and so could contribute in distinct manners to disease susceptibility and, consequently, should be addressed separately [59,60]. A meta-analysis using the entire data was also carried out to weigh and summarize the results obtained with the multiple single TDT tests. The results are shown for each population and for the pooled meta-analysis in Figure 2. Meta-analysis forest plots were constructed for each marker (data not shown) and the six most significant markers from the overall meta-analysis are illustrated in Figure 3. No significant findings were obtained with the *LRRTM1 *gene analysis. One marker (rs1488454) in *LRRN1 *showed statistically significant transmission disequilibrium, having a preferential transmission of the C allele to the affected offspring (*P *= 0.002 - Additional File 1 and Figure 2). This significance was present in the singleton cohort within the IMGSAC subgroup, as confirmed in the corresponding forest plot where this population shows an OR deviation from 1 (Figure 3a). For *LRRN3*, the marker rs10261004 was significant in the Northern Dutch cohort (*P *= 0.001 - Additional File 2 and Figure 2). This is shown in the respective forest plot for this subgroup (Figure 3c), with the condition less likely in individuals who carry the G allele (as the OR is below 1). Moreover, the two neighbouring markers were also of nominal significance in the same population. However, this significance was not present after adjusting for multiple testing corrections. In *LRRTM3*, two SNPs - rs1998753 (*P *= 0.001) and rs12266823 (*P *= 0.001 - Additional File 3 and Figure 2) - showed statistically significant transmission disequilibrium. For these markers the significance was being driven especially by the multiplex cohort. However, the pooled meta-analysis was also significant for rs12266823 (Figure 2), showing that other groups were also contributing to the association in the same direction. Furthermore, the respective forest plots (Figure 3d-e) show that the G allele for rs1998753 confers an increased risk for ASD, while the A allele for rs12266823 confers a decreased risk. Additionally, rs10997476 had nominal significance, especially in the multiplex samples (Figure 3f), but the significance did not remain after we adjusted for multiple testing. The empirical *P*-values for all TDTs performed are reported in Additional Files 1 to 6, and the significant ones are highlighted in bold. Parental transmissions were examined for each marker, but no evidence of parent-of-origin effects was found (*P *> 0.12).

Examination of the LD patterns across *LRRN1*, *LRRN3 *and *LRRTM3 *loci, using our genotyping data, showed that each gene defined four, two and four LD blocks, respectively (r^2 ^> 0.8 and MAF > 0.05; Figure 1). In contrast, the SNPs tested in *LRRTM1 *defined no LD blocks. 'The human genome can be parsed objectively into haplotype blocks: sizable regions over which there is little evidence for historical recombination and within which only a few common haplotypes are observed' [61], which I will call LD blocks. 'If haplotype blocks represent regions inherited without substantial recombination in the ancestors of the current population, then a biological basis for defining haplotype blocks is to examine patterns of recombination across each region. The history of recombination between a pair of SNPs can be estimated with the use of the normalized measure of allelic association, D' '[61]. 'We define pairs to be in "strong LD" if the one-sided upper 95% confidence bound on D' is >0.98 (that is, consistent with no historical recombination) and the lower bound is above 0.7' [61]. Haplotype analysis was performed in order to further assess the transmission disequilibrium within the LD blocks, as haplotypes can sometimes offer more power to detect association compared to single SNPs. Transmission of haplotypes, including all markers within each block, was tested using Transmit [56], along with the multi-marker haplotype combinations from the Tagger output (since the haplotype tagging SNPs were chosen using aggressive tagging). In the overall population, only one significant haplotype specific result was obtained, for a Tagger multi-marker haplotype within *LRRTM3 *(Table 1). This haplotype contains marker rs12266823 that showed high significance in the single locus association test. Additionally, the two markers in intron 2 (rs6480245 and rs12266823) that comprise this haplotype are in high LD with each other (D' = 87 - Figure 1c). However, for haplotype GA (*P *= 0.0005 - Table 1) the significance of the haplotype was increased compared to the single marker, suggesting that this specific haplotype could potentially increase susceptibility to ASD. Two three-marker haplotypes (rs716546-rs716547-rs9971243) were also over-transmitted to the affected offspring, revealing a possible transmission distortion in LD block 2. However, the significance was above the 0.005 significance threshold established previously and none of the markers that make the haplotypes are in high LD with the significant markers from the single locus TDT test. The same analysis was carried out for separately each gene across the five subgroups of samples and only the haplotype specific *P*-values approaching or below the nominal significance threshold of *P *< 0.05 are shown (Table 2). No significant results were obtained with the German and Italian populations or with *LRRTM1*. Taking each population into account, although some results reach nominal significance for *LRRN1 *and *LRRTM3*, they were all above the 0.005 significance threshold previously established. However, for the Northern Dutch population, *LRRN3 *showed a transmission distortion in LD block 2 with the two-marker (rs10261004 and rs214884) haplotypes GC (*P *= 0.005 - Table 2) and TT (*P *= 0.004 - Table 2). However, these haplotypes both contain rs10261004 which, alone, was significant and is most probably driving the association in this case, as the significance was not increased compared to the single locus result.

**Table 1:** Haplotype transmission disequilibrium results in the overall population across leucine-rich repeat transmembrane neuronal *(LRRTM3)*.

LD block/Tag^b^	Haplotype^a^	TR	NT	OR	CI	*P*-value
Block 2 (rs716546-rs716547-rs9971243)	CCT	75	52	1.44	(1.01, 2.05)	0.0505
Block 2 (rs716546-rs716547-rs9971243)	CTC	2	10	0.20	(0.04, 0.91)	0.0386
rs6480245, rs12266823^*b*^	GA	44	84	0.52	(0.36, 0.75)	0.0005
rs6480245, rs12266823^*b*^	GC	242	186	1.30	(1.07, 1.58)	0.0078

The analysis was performed within each linkage disequilibrium (LD) block for each gene and for the haplotype combinations from Haploview (selected by Tagger). Only haplotypes approaching or below the nominal significance threshold of *P *< 0.05 are shown.

^a^Haplotypes only reported if there are more than 10 informative transmissions in total.

^***b***^Haplotype combinations selected by Tagger.

CI, confidence interval; NT, non-transmitted; OR, odds ratio; SNP, single nucleotide polymorphism; TR, transmitted.

**Table 2:** Haplotype transmission disequilibrium results per population across the four genes studied.

Gene	LD block/Tag^b^	Hap^a^	*IMGSAC *(Multiplex families)				*IMGSAC *(Singleton families)				*Northern Dutch*			
			
			TR	NT	OR/CI	*P*-value	TR	NT	OR/CI	*P*-value	TR	NT	OR/CI	*P*-value
***LRRN3***	Block 2 (rs10261004-rs214884)	GC									9	26	0.35 (0.16, 0.74)	0.005
***LRRN3***	Block 2 (rs10261004-rs214884)	TT									44	20	2.20 (1.30, 3.73)	0.004

***LRRN1***	Block 3 (rs10510278-rs696489)	AC	98	70	1.40 (1.03, 1.90)	0.037								
***LRRN1***	Block 3 (rs10510278-rs696489)	TT	61	85	0.72 (0.52, 1.00)	0.057								
***LRRN1***	rs10780025, rs13100776^***b***^	AT	18	37	0.49 (0.28, 0.85)	0.014								

***LRRTM3***	rs1925613, rs10762138^***b***^	CT	8	20	0.40 (0.18, 0.91)	0.036								
***LRRTM3***	rs1925613, rs1925624^***b***^	AT	139	102	1.36 (1.06, 1.76)	0.020								
***LRRTM3***	rs2619652, rs12785206^***b***^	CC					18	7	2.57 (1.07, 6.16)	0.043				
***LRRTM3***	rs2619652, rs12785206^***b***^	TT					9	22	0.41 (0.19, 0.89)	0.029				
***LRRTM3***	rs1925613, rs12785206^***b***^	CT					8	21	0.38 (0.17, 0.86)	0.024				

^The analysis was performed within each linkage disequilibrium (LD) block for each gene and for the haplotype combinations from Haploview (selected by Tagger). Only haplotypes^a ^approaching or below the nominal significance threshold of *P *< 0.05 are shown.^

^a^Haplotypes only reported if there are more than 10 informative transmissions in total.

^***b***^Haplotype combinations selected by Tagger.

CI, confidence interval; Hap, Haplotype; NT, non-transmitted; OR, odds ratio; SNP, single nucleotide polymorphism; TR, transmitted.

### Case-control study

**Figure 4:**
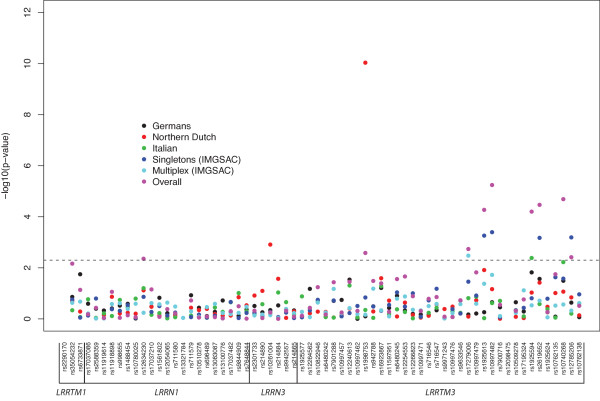
**Case-control allele-based tests of association performed for each population and for the overall meta-analysis**. The significance is represented on the *y*-axis, plotted as -log_10_(*P*-value) and on the *x*-axis is shown each marker studied. The dashed line represents the statistical significance threshold of 0.005. 'Overall' represents the pooled meta-analysis performed.

A case-control study was conducted in parallel, testing probands with ASD against unselected controls, using allele and genotype data from the same 66 SNPs genotyped across the four LRR genes. Minor allele frequencies from controls and cases for each population across the genes under study were calculated and the SNPs were in Hardy-Weinberg equilibrium in both (*P *> 0.001). Case-control analysis was carried out using logistic regression in probands of each population against the 295 ECACC controls. Results for the allelic tests of association applied to each sample cohort and for the overall meta-analysis are shown in Figure 4 and, again, *P *< 0.005 were considered significant. In order to complement and further describe the latter, the most significant results for the genotypic tests of association performed are shown in Table 3. The allelic case control analysis highlighted nine SNPs in total. One marker in *LRRN3 *(rs10261004) and one in *LRRTM3 *(rs1998753) were again significant, confirming the results of the single *locus *TDT study. For rs10261004 the association was detected in the Northern Dutch cohort (consistent with earlier results) and tests of association indicated a decreased risk for the G allele compared with the T (G versus T: *P *= 0.001; additive G: *P *< 0.003). The rs1998753 association is being driven by the Northern Dutch cohort (A versus G: *P *< 0.00001; AG versus GG/AA: *P *< 0.00001), with the other cohorts all having non-significant ORs less than 1 (A versus G: *P *= 0.21; AG versus GG: *P *= 0.48). Across all populations there were no 'AA' genotypes for rs1998753 and the minor allele frequencies were low (A allele: <5%), except in the Northern Dutch cohort (A allele: MAF 0.234), consistent with family-based frequencies. Additionally, there were seven new significant results (*P *< 0.005) located in *LRRTM3 (*rs17279006, rs1925613, rs10997482, rs1925594, rs2619652, rs10740268 and rs12785206; Figure 4). The first three polymorphisms (rs17279006, rs1925613, rs10997482) were found to be significant in the overall meta-analysis. The overall results of association tests were: rs17279006 (G versus A: *P *< 0.002; additive G: *P *< 0.003), rs1925613 (C versus A: *P *< 0.00006; additive C: *P *< 0.00008) and rs10997482 (G versus A: *P *< 0.000006; additive G: *P *< 0.00001). The markers rs1925613 and rs10997482 were most significant in the singleton cohort (rs1925613, C versus A, *P *< 0.0006; additive C, *P *< 0.0009; and rs10997482, G versus A, *P *= 0.0004; additive A: *P *< 0.0007). For rs1925594, there was a trend towards increased risk for the C allele compared with the T in the Italian population and in the meta-analysis. However, the number of allele counts is too low to reach a confident conclusion about this result. The marker rs2619652 was significant in the overall meta-analysis and, in particular, in the singleton cohort, presenting an increased risk for the C allele compared with the T (*P *< 0.0007; additive T: *P *< 0.002). The marker rs10740268 was only significant in the overall meta-analysis, showing an increased risk for the C allele compared with T (*P *< 0.00002; additive C: *P *< 0.00003). Lastly, the marker rs12785206 was, again, more significant in the singleton cohort, having a decreased risk of the C allele compared with the T (*P *< 0.0007; CC/CT versus TT: *P *= 0.0004). Overall, the case-control provides increasing evidence of significant association between *LRRTM3 *and ASD susceptibility, not only by confirming the association with rs1998753 but also through the new significant results within the gene.

**Table 3:** Case-control genotype-based test results.

*SNP*	*Population*	*Model*	*OR*	*CI*	*P-value*
rs10261004	Northern Dutch	Additive G	0.44	(0.26, 0.75)	3.00E-03
rs10261004	Northern Dutch	TT versus GG/GT	2.42	(1.33, 4.41)	4.00E-03
rs1998753	Northern Dutch	Additive G	0.10	(0.05, 0.20)	5.68E-12
rs1998753	Northern Dutch	AG versus GG/AA	9.64	(5.06, 18.38)	5.68E-12
rs17279006	Overall	Additive G	0.62	(0.45, 0.84)	2.22E-03
rs17279006	Overall	AA versus GG/AG	1.65	(1.19, 2.29)	2.59E-03
rs17279006	Multiplex (IMGSAC)	AA versus GG/AG	2.14	(1.28, 3.59)	3.96E-03
rs17279006	Multiplex (IMGSAC)	Additive G	0.49	(0.30, 0.79)	4.00E-03
rs1925613	Overall	CC versus AA/AC	1.81	(1.37, 2.39)	2.97E-05
rs1925613	Overall	Additive C	1.35	(1.16, 1.57)	7.82E-05
rs1925613	Singleton (IMGSAC)	CC versus AA/AC	3.01	(1.66, 5.47)	2.81E-04
rs1925613	Singleton (IMGSAC)	Additive C	1.80	(1.27, 2.54)	8.97E-04
rs10997482	Overall	Additive G	1.38	(1.20, 1.60)	9.81E-06
rs10997482	Overall	GG versus AA/AG	1.60	(1.27, 2.00)	4.63E-05
rs10997482	Singleton (IMGSAC)	Additive A	0.54	(0.38, 0.77)	6.85E-04
rs10997482	Singleton (IMGSAC)	GG versus AA/AG	2.41	(1.45, 4.03)	7.45E-04
rs2619652	Overall	Additive T	0.76	(0.67, 0.88)	1.57E-04
rs2619652	Overall	TT versus CC/CT	0.63	(0.49, 0.81)	2.71E-04
rs2619652	Singleton (IMGSAC)	Additive T	0.58	(0.41, 0.82)	1.95E-03
rs2619652	Singleton (IMGSAC)	TT versus CC/CT	0.34	(0.17, 0.69)	2.56E-03
rs10740268	Overall	TT versus CC/CT	0.51	(0.38, 0.70)	2.16E-05
rs10740268	Overall	Additive C	1.84	(1.38, 2.45)	3.48E-05
rs12785206	Overall	TT versus CC/CT	1.49	(1.20, 1.86)	3.96E-04
rs12785206	Singleton (IMGSAC)	CC/CT versus TT	0.37	(0.22, 0.65)	4.20E-04
rs12785206	Singleton (IMGSAC)	Additive C	0.48	(0.31, 0.74)	1.00E-03

Only the most significant results are shown (*P *< 0.005).  SNP, single nucleotide polymorphism; OR, odds ratio; CI, confidence interval.

## Discussion

Recently, there has been increasing attention towards the LRR group of transmembrane proteins and their relationship with neurological complex disorders. The LRRs analysed here are thought to be synaptic organizing molecules and have been implicated in brain development, which is often impaired in autistic individuals [8,14,18,19,21,32]. In the current study, we hypothesized that common variants in the leucine motifs gene class could possibly be involved in ASD pathogenesis. Specifically, *LRRTM1*, *LRRTM3*, *LRRN1 *and *LRRN3 *were examined as candidate genes in four different populations of European ancestry by carrying out parallel family-based and case-control studies.

*LRRN1 *showed only one significant result with the marker rs1488454, showing that its C allele is increasing the susceptibility to ASDs in the singleton cohort. However, this result was not confirmed by the case-control analysis which points to a weaker evidence for a role in susceptibility.

The family based study showed that rs10261004 within *LRRN3 *is strongly associated with ASD risk in the Northern Dutch cohort and that its G allele is protective for susceptibility to the disorder. The haplotype analysis also showed evidence of significance with two haplotypes within LD block 2. Moreover, the case-control analysis confirmed the evidence of an association with this marker, showing a decreased risk for the G allele and for the GG/GT genotypes (compared to the T allele and TT genotype, respectively). This is the most consistent result that we have found, as the marker is significant in all the tests performed in the Northern Dutch cohort and, therefore, it could be a population specific susceptibility. The non-association reported for this gene in the Collaborative Linkage Study of Autism dataset by Hutcheson *et al*. [45] could be explained by several factors: differences in sample ascertainment, family numbers and the hypothesis that one of the results is a false positive. In effect, they tested 30 nuclear families, which could have limited their ability to detect the association signal that we are observing in our cohort. In addition, none of the four markers tested in their study were in high LD with rs10261004.

*LRRTM3 *was the gene that showed most evidence of association in the populations that we studied. In particular, rs1998753 was the most consistent significant marker within this gene, showing that its G allele increases the risk for ASD, especially in the multiplex cohort (but was also significant in the pooled meta-analysis). Although this effect was evident in all case-control cohorts except the Northern Dutch, it did not reach a significant level of statistical evidence. In fact, in the Northern Dutch case-control cohort, which is a more isolated population than the others, there is strong evidence that the A allele increases the risk for ASD and that there is a non-significant over-transmission of the A allele to cases in the TDT. Consequently, for the case-control, although the Northern Dutch result could be a false positive, it biases the overall results. The statistical power of the case-control analysis is hindered by the small sample size of our control cohort, by sampling cases from a population possibly different to the others and by false positives which were possibly due to the population structure. In contrast, the family-based study is robust to population stratification. So, although the case-control result can be taken as a verification of the positive evidence of association with this marker, this result warrants further confirmation that could be achieved by another replication study or by genotyping a larger control group for this marker, preferably including a set of controls from the Northern Dutch population. In addition to rs1998753, rs12266823 was also significant, with the A allele being protective against ASD risk in the multiplex cohort and in the pooled meta-analysis of the TDT. However, this result was not confirmed by the case-control analysis. Nevertheless, new associations within *LRRTM3 *were found in the case-control study with the markers rs17279006, rs1925613, rs10997482, rs2619652, rs10740268 and rs12785206. All of these were significant in the overall analysis. For rs17279006 the G allele and GG(/GT) genotypes were protective against the disorder, whereas for the rs1925613 C allele, and CC(/CA) and rs10997482 G allele and GG(/GA) genotypes, the risk of ASD was increased (the last two markers being most significant in the singleton cohort in the genotypic test). For rs10740268 the C allele and CC(/CT) genotypes there was an increasing risk to ASDs in the overall meta-analysis. Moreover, rs2619652 and rs12785206 were both significant in the singleton cohort; the first presenting an increased risk for the C allele and CC (/CT) genotypes and the second having a decreased risk for the C allele and CC/CT genotypes. Therefore, overall there is growing evidence that *LRRTM3 *may play a role in ASD susceptibility.

Interestingly, significant results from the TDT analysis were only replicated in the case-control study for two markers. However, seven new significant markers appeared in the latter that were not previously identified by the TDT. Several explanations can be proposed for this occurrence but the most evident is the use of different analytical approaches. Both TDT and case-control studies use different methods to access association, and while the TDT is more robust to population stratification, the case-control has in general more power to detect association in studies such as ours. We cannot exclude the possibility that subtle population structure (detected using many hundreds or thousands of markers) may lead to false positive results in the case-control analyses above. We suggest further that there should be an assessment of the case-control specific association markers in a larger number of controls and, if possible, a correction for population structure or other confounding effects using many more markers. The two analyses presented complement each other and reflect the complex heterogeneity of the disorder spectrum, pointing once more to the difficulty of studying ASDs. Additionally, different markers within *LRRTM3 *were differently associated to multiplex and singleton families, supporting the theory that we are looking at genetically different groups within the same population that have different patterns of ASD traits [59,60].

As both associated genes, *LRRTM3 *and *LRRN3*, are nested, they are possibly transcriptionally co-regulated with the genes that contain them (*CTNNA3 *and *IMMP2L*, respectively) [8]. Unknown regulatory mechanisms could be present in these regions altering gene expression or even splicing patterns which, in turn, could contribute to ASD susceptibility. Moreover, Wang *et al*. found an association with autism with one intronic marker (rs9651325) in *CTNNA3*, which is located 3' to *LRRTM3*, a region that was not covered by our study [13]. Both these LRR genes warrant further investigation as they are highly expressed in the brain, particularly the cerebellum, [8,14] (a characteristically impaired region in autistic individuals [62,63]), involved in central nervous system development and regeneration [8,14,27,28,31], and there is accumulating evidence implicating these genic regions in ASD susceptibility [13,44].

## Conclusions

Taken together, there is converging evidence that common genetic variants in *LRRTM3 *and *LRRN3 *confer susceptibility to ASD and further study of these genes and their function will provide valuable insights into their role in ASD pathogenesis. In summary, this is one of the first studies to show results of an association between more than one leucine-rich repeat gene and ASD susceptibility in populations of European ancestry.

## List of Abbreviations

ASDs: autism spectrum disorders; ADI-R: autism diagnostic interview-revised; ADOS: autism diagnostic observation schedule; CI: confidence interval; CNS: central nervous system; *CTNNA2*: *α2-cateni; CTNNA3*: *αT-catenin; *ECACC: European Collection of Cell Cultures; IMGSAC: The International Molecular Genetic Study of Autism Consortium; *IMMP2L*: inner mitochondrial membrane peptidase-like; LD: linkage disequilibrium; LRR: leucine-rich repeat; LRRN: leucine rich repeat neuronal; LRRTM: leucine rich repeat transmembrane neuronal; MAF: minor allele frequency; mRNA: messenger RNA; OR: odds ratio; SNP: single nucleotide polymorphism; TDT: transmission disequilibrium test.

## Competing interests

The authors declare that they have no competing interests.

## Authors' contributions

IS, TGC and APM participated in the design of the study. IS performed the molecular genetic study (acquisition, statistical analysis and interpretation of the data) and carried out the writing of the manuscript. TGC performed the statistical analysis as well as the interpretation of the data. RH and ATP both helped to perform the DNA preparation, primer selection and interpretation of the data, as well critically revising the manuscript for intellectual content. EM, RBM, AJB, AB, SMK and FP performed the clinical assessment of patients and DNA sample collection of the Northern Dutch, IMGSAC, Italian and German populations, as well as revising for intellectual content. APM supervised the molecular genetic experiments and critically revised the manuscript for intellectual content. All authors read and approved the final manuscript.

## Supplementary Material

Additional file 1 TDT results for the single nucleotide polymorphisms genotyped across *LRRTM1*, *LRRN1*, *LRRN3 *and *LRRTM3 *genes in the singleton families of the International Molecular Genetic Study of Autism Consortium population. Click here for file

Additional file 2 TDT results for the single nucleotide polymorphisms genotyped across *LRRTM1*, *LRRN1*, *LRRN3 *and *LRRTM3 *genes in the Northern Dutch population. Click here for file

Additional file 3 TDT results for the single nucleotide polymorphisms genotyped across *LRRTM1*, *LRRN1*, *LRRN3 *and *LRRTM3 *genes in the multiplex families of the International Molecular Genetic Study of Autism Consortium population. Click here for file

Additional file 4 TDT results for the single nucleotide polymorphisms genotyped across *LRRTM1*, *LRRN1*, *LRRN3 *and *LRRTM3 *genes in the Italian population. Click here for file

Additional file 5 TDT results for the single nucleotide polymorphisms genotyped across *LRRTM1*, *LRRN1*, *LRRN3 *and *LRRTM3 *genes in the German population. Click here for file

Additional file 6 TDT Meta-analysis results for the single nucleotide polymorphisms genotyped across *LRRTM1*, *LRRN1*, *LRRN3 *and *LRRTM3 *genes. Click here for file
